# Multimodal Optical Monitoring of Auto- and Allografts of Skin on a Burn Wound

**DOI:** 10.3390/biomedicines11020351

**Published:** 2023-01-26

**Authors:** Ilya Turchin, Vladimir Beschastnov, Petr Peretyagin, Valeriya Perekatova, Alexey Kostyuk, Anna Orlova, Nikita Koloshein, Aleksandr Khilov, Ekaterina Sergeeva, Mikhail Kirillin, Maksim Ryabkov

**Affiliations:** 1 Institute of Applied Physics of Russian Academy of Sciences, 46 Ulyanov St., 603950 Nizhny Novgorod, Russia; 2 University Clinic, Privolzhsky Research Medical University, 10/1 Minin and Pozharsky Sq., 603950 Nizhny Novgorod, Russia

**Keywords:** skin allografts, skin autografts, burn wound, optical diffuse spectroscopy, diffuse reflectance spectroscopy, laser Doppler flowmetry, optical coherence tomography, oxygenation, blood perfusion

## Abstract

The aim of the study was to investigate the dynamics of the state of allo- and autografts of skin on a wound using optical modalities: diffuse reflectance spectroscopy (DRS), optical coherence tomography (OCT), and laser Doppler flowmetry (LDF). A deep thermal burn was simulated in 24 rats covering 20% of the body surface. On day 3 after the injury, a fascial necrectomy of two 500 mm^2^ areas on the left and right sides of the midline of the animal body were excised. Allografts and autografts were placed in the centers of these areas. Optical measurements of grafts were performed on the 0, 3rd, 6th, 10th, and 13th days after transplantation. The allografts demonstrated a pronounced decrease in oxygenation, blood content, and perfusion compared to autografts on the 6th day; in the following days of observation, these values returned to the average values of autografts. Water content gradually decreased from the beginning to the end of observation. In conclusion, optical diagnostics revealed changes in the morphological microstructure, the rate of restoration of blood circulation, and oxygen exchange in the early stages, specific for the allo- and autograft.

## 1. Introduction

Modern approaches for deep skin burn treatment are supposed to use split-thickness skin grafts (STSG). In patients with subcritical and critical areas of burns, autografts from the patient skin and allografts from the donor skin are used [1,2]. Artificial dermal substitutes (DSS) have prospects as a modern alternative to auto- and allografts of the skin. DSS are fundamental in physiological wound healing to ensure consistent and enduring wound closure and provide a suitable scaffold to repair tissue [3]. However, in terms of several clinically significant characteristics (cost, resistance to infection, etc.), many DSS are still inferior to natural skin grafts and cannot completely replace them.

Allografts can serve as temporary wound coverage until the patient’s condition allows autodermoplasty or can be used simultaneously with autoplasty as a protector for autologous grafts [4,5]. Simultaneous use of autologous and allogeneic skin grafts is complicated by the different ways of the wound repair processes. Allografts serve as a protector for several days, with the following rejection due to contact with the recipient’s immune system, and can cause severe local and general complications [6]. Early removal of the allograft will reduce clinical efficacy, but an accurate prediction of the rejection time is still challenging [7]. In contrast to the allograft, an autograft is not rejected and should be kept on the wound as long as possible until complete engraftment. It is important to note that the trigger for the onset of allograft rejection and the pathogenetic basis for engraftment of the autograft is the same process: the inclusion of grafts in the blood and lymph circulation of the recipient. This fact explains the importance of studying the dynamics of blood perfusion in grafts.

Standard medical imaging modalities, MRI and US, can be applied to assess the vascular bed and tissue microstructure of heart and kidney allografts [8,9]. However, MRI and US have some limitations in application to skin graft diagnostics [10], and optical modalities have shown good potential in plastic surgery of the skin, providing information on the functional or structural state of biological tissue. Laser Speckle Contrast Imaging was used to monitor reperfusion of free full-thickness skin grafts [11], and Laser Doppler Imaging as well as Laser Doppler Flowmetry may assess burn depth and predict the need for excision and grafting [12,13]. Optical coherence tomography (OCT) and Optical Coherence Angiography (OCA) allow direct visualization of tissue structure and capillary-level vascular structure within the skin [14], and were applied for pediatric heart transplant recipients [15]. Diffuse reflectance spectroscopy (DRS) in the near-infrared wavelength range has been proposed to determine the depth of burn wounds [16,17,18]. Wide-field diffuse optical imaging showed its potential for long-term investigation of the efficacy of tissue healing. It revealed a temporary increase in blood content 1 week after treatment of a burn using the STSG [19].

However, the problem of early non-invasive diagnosis of split skin allograft rejection has not been solved so far. The aim of this study is to investigate the state of auto- and allografts of the skin in a burn wound using different optical modalities, DRS, OCT/OCA, and LDF, which provide complementary information about the tissue state [20]. While DRS gives information on the physiological status of biological tissue from chromophore content (blood and water content and oxygenation), OCT provides information on tissue structure, and OCA and LDF provide data on blood perfusion.

## 2. Materials and Methods

### 2.1. Animal Preparation

**Figure 1:**
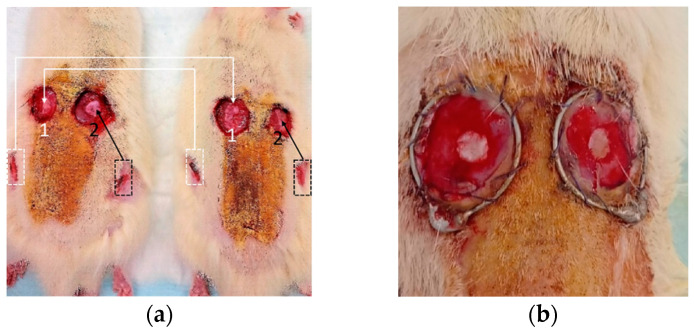
Photos of a deep thermal burn simulation in rats. Two rounded areas correspond to fascial necrectomy. In the area (1), an allodermal graft was placed, and in the area (2), an autograft was placed (**a**). Image (**b**) presents these areas with higher magnification.

The animal studies were approved by the Ethics Committee of Privolzhsky Research Medical University (protocol no. 5, 10.03.2021). An experimental study was carried out on outbred Wistar rats weighing 255–380 g (males); *n* = 24 rats were selected from different litters, and they were not related. A deep thermal burn was simulated on the back surface of a rat with an area of 20% of the body surface (Figure 1) corresponding to the III stage. Three days after the injury, a partial fascial necrectomy was performed: two rounded areas of 500 mm^2^ square were excised to the right and left of the midline. In each animal, an autodermal graft with an area of 50 mm^2^ and a thickness of 0.5 mm was placed in the center of the wound, localized to the right of the midline; an allodermograft of the same size was placed on the wound located to the left of the midline. The wounds were closed with an aseptic wet dressing. 

The procedures were performed under general anesthesia with a mixture of solutions of 3.5% tiletamine hydrochloride and 2% xylazine hydrochloride, which was administered intramuscularly to the animals.

### 2.2. Skin Engraftment Research

At the control dates (3rd, 6th, 10th, and 13th days after transplantation), the dressings were removed and the condition of the micrografts were examined using LDF, OCT, and DRS (Table 1). At each control stage, six animals were taken out of the experiment, and allo- and autografts were taken for histological examination.

### 2.3. Diffuse Reflectance Spectroscopy (DRS)

**Figure 2:**
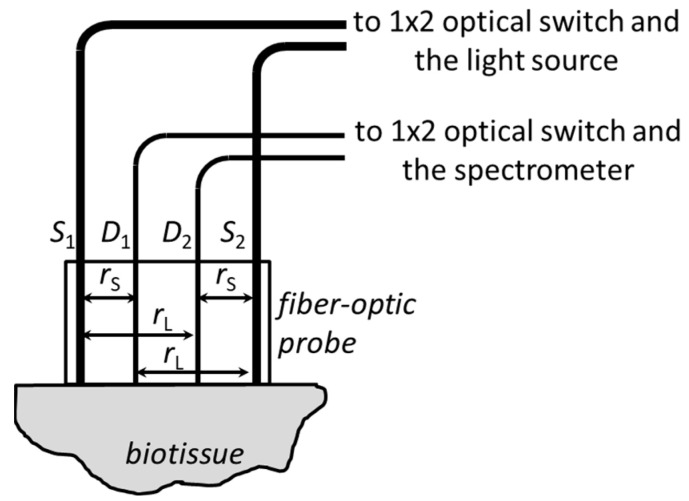
Fiber-optic probe design in DRS experimental setup employing a self-calibrating approach. S1 and S2 are the source fibers, D1 and D2 are the detection fibers.

DRS measurements of tissue blood and water content and oxygenation were performed using the experimental setup constructed at the Institute of Applied Physics (Nizhny Novgorod, Russia), which has a contact fiber-optic probe for delivering light on a tissue and collecting backscattered light. The probe uses two 400-µm sources (S1, S2) and two 200-µm detection fibers (D1, D2) positioned in line with equal distances of 2 mm between neighbor fibers (Figure 2). Short $(r_{s}=2\;$ mm) and long ($r_{L}=4$ mm) source detector distances (SDD) are available in this probe for each source fiber S1 and S2. 

Four measurements (S1-D1, S1-D2, S2-D1, S2-D2) were performed to apply the self-calibrating approach [21], which gives robust data on the extinction coefficient spectrum $\mu_{eff}$ in tissue. In the frames of diffusion, an approximation of the Radiative Transfer Equation [22] extinction coefficient can be expressed from the measured data as:

(1) $$\mu_{eff}=\sqrt{3\mu_{a}\left(\mu_{a}+\mu_{s}^{\prime }\right)}$$

(2) $$\mu_{eff}=\frac{1}{2\left(r_{L}-r_{S}\right)}\ln\left[\left(\frac{I_{11}I_{22}}{I_{12}I_{21}}\right)\left(\frac{E_{12}E_{21}}{E_{11}E_{22}}\right)\frac{r_{S}^{2}}{r_{L}^{2}}\right]$$

where ${\;\mu }_{a}$ and $\mu_{s}^{\prime }$ are absorption and the reduced scattering coefficients of the investigated object; $I_{ij}$ are the registered backscattered spectra subtracted by the background for each source–detector combination (indices i=1,2 and j=1,2 are the source and detector numbers correspondingly); and $E_{ij}$ are the corresponding integration times for each measurement. The pair of measurements $I_{11}$ and $I_{22}$ correspond to short SDD, and the pair $I_{12}$ and $I_{21}$ correspond to long SDD. 

Each source fiber is connected to the Fiber-Coupled Xenon (SLS205, Thorlabs, USA) 240–1200 nm lamp via a 1x2 fiber-optic switch (Piezosystem Jena GmbH, Germany). Each detection fiber is connected to the Maya 2000 PRO (Ocean Optics, USA) spectrometer via a 1x4 fiber-optic switch (Piezosystem Jena GmbH, Germany). To optimally fit the dynamic range of a spectrometer, individual integration times were used for short SDD $E_{11}=E_{22}=15\;\mathrm{ms}$ and long SDD $E_{12}=E_{21}=80\;\mathrm{ms}$. 

The applied combination of the light source, spectrometer, SDDs, and integration times gives a high enough signal-to-noise ratio of the registered spectral data $I_{ij}$ in the range of 460–1030 nm for the rat skin. This spectral range allows reconstruction of oxy- and deoxyhemoglobin concentrations both in the visible (500–600 nm) and near-infrared (700–900 nm) ranges, and the water content near the absorption peak of 975 nm.

The simplified model functions of absorption and the reduced spectra of a “generic” tissue [23] were used to reconstruct the blood content, $C_{blood}$, blood saturation, $StO_{2}$, and water content, $C_{water}$, from the measured extinction spectrum (2):

(3) $$\begin{array}{ll} \mu_{a}(\lambda )= & C_{water}*\mu_{a}^{water}(\lambda )+C_{mel}*\mu_{a}^{mel}(\lambda )+\\ & \left[C_{blood}*\left(StO_{2}\;*\mu_{a}^{oxy}(\lambda )+\left(1-StO_{2}\right)*\mu_{a}^{deoxy}(\lambda )\right)\right]+\mu_{a}^{dry}, \end{array}$$

(4) $$\mu_{s}^{\prime }\left(\lambda \right)=a{\left(\frac{\lambda }{\lambda_{0}}\right)}^{-b}+c,$$

where $\mu_{a}^{water}$, $\mu_{a}^{mel}$, $\mu_{a}^{oxy}$, and $\mu_{a}^{deoxy}$ are the partial spectra of water, melanin, oxy-, and deoxyhemoglobin taken from [24,25]; $\mu_{a}^{dry}$ is an absorption of a “dry matter” taken as a wavelength-independent value; *a, b* and *c* are the values characterizing the reduced scattering spectrum [23], and $\lambda_{0}=550\;\mathrm{nm}$. The sought values of $C_{blood}$, $StO_{2}$, and $C_{water}$ are reconstructed along with other free parameters $\mu_{a}^{dry}$, *a*, *b,* and *c* using lsqcurvefit MATLAB minimization function giving the best fit for the measured extinction spectrum:

(5) $$\sum_{\lambda }{\left(\sqrt{3\mu_{a}\left(\mu_{a}+\mu_{s}^{\prime }\right)}-\frac{1}{2\left(r_{L}-r_{S}\right)}\ln\left[\left(\frac{I_{11}I_{22}}{I_{12}I_{21}}\right)\left(\frac{E_{12}E_{21}}{E_{11}E_{22}}\right)\frac{r_{S}^{2}}{r_{L}^{2}}\right]\right)}^{2}\underset{\begin{array}{c} C_{blood},\;StO_{2}\\ C_{water},\mu_{dry},a,\;b,c \end{array}}{\to }\min,$$

where $\Sigma_{\lambda }$ represents the sum over all wavelengths of a spectrometer in the range of 460–1030 nm. The melanin concentration is assumed constant equal to $C_{mel}=0.005$, because ${\;\mu }_{a}^{\mathrm{mel}}(\lambda )$ and $\mu_{s}^{\prime }(\lambda )$ both monotonously decrease with the wavelength, and a high uncertainty arises in the joint reconstruction of the parameters of $C_{mel}$, *a*, *b,* and *c*.

The DRS measurements were performed for normal skin and the skin grafts in control dates according to Table 1. Each measurement was repeated three times, and the values of $C_{blood}$, $StO_{2}$, and $C_{water}$ were averaged over reconstructed results. The results of reconstruction were not included in the averaging procedure if the root-mean-square error (RMSE) between experimental and fitting curves is more than 0.05, which indicates the high discrepancy between the measured and the model functions.

### 2.4. Laser Doppler Flowmetry (LDF) 

An LDF Lasma-MC (LLC NPP LAZMA, Russia) device was used to assess microcirculation in micrografts. The following indicators of basal blood flow were obtained using LDF: microcirculation index, PM, and the variable component of the signal as the standard deviation of perfusion fluctuations, δPM. To assess the mechanism of reperfusion of micrografts, we compared the contribution of active microcirculation control factors in the frequency range of 0.005–0.12 Hz, which corresponds to endothelial, neurogenic, and myogenic factors of the intrinsic activity of the vascular wall, and passive control factors in the range of 0.2–1.6 Hz, which corresponds to external components of the regulation of respiratory and cardiac blood flow.

### 2.5. Optical Coherence Tomography (OCT) 

Structural and functional analysis of the micrografts was performed using OCT-1300E device (IAP RAS, Biomedtech Ltd., Nizhny Novgorod, Russia) for optical coherence tomography (OCT) combined with OCT-angiography (OCT-A) with a probing wavelength of 1300 nm. The device is equipped with a rigid probe of 1 cm in diameter and provides a spatial resolution of 15 µm with a probing depth of up to 1.5 mm. The scan acquisition rate is 20000 A-scans per second resulting in 26 s for the acquisition of a 3D volume of 3 × 3 × 1.5 mm^3^ collocated with an angiography map. Both structural and angiographic OCT images were acquired at each time point for each available animal. Since the area of the OCT probe is comparable to the entire area of the graft, its position was selected to maximally cover the graft area (although the imaging area of OCT is only 3 × 3 mm^2^ and corresponds to the central area of the probe). The probe was situated to maximally provide contact of the probe surface with the tissue surface to ensure better acquisition of the OCT-angiography image.

To perform a formal analysis of the registered OCT data, we developed a three-grade semi-quantitative scoring system to estimate the manifestation of edema and microcirculatory activity based on OCT-angiography data (Table 2). This table describes the main features of the OCT data that allow attributing an image with a score.

### 2.6. Histological Examination

A biopsy of the auto- and allograft with adjacent wound tissues was performed in four stages: in six animals (group I), the biopsy was taken on the 0th day, in six (group II) on the 3rd day, in six (group III) on the 10th day, and in the remaining six (group IV) on the 13th day after transplantation. The tissue was fixed in 10% formalin in phosphate buffer (pH 7.2), then subjected to standard histological processing and embedded in HISTOMIX-extra paraffin-based embedding medium (Biovitrum, St.-Petersburg, Russia) or TissuePrep 2 (Fisher Scientific, Waltham, MA, USA). On a Leica SM 2000 R microtome, histological sections of the central region of the wounds were made in the transverse plane with a thickness of 5 μm. For morphological examination, slices were stained with hematoxylin-eosin. Preparations were studied using a Nikon Eclipse 80i microscope, the microscopic images were captured with a Nikon Ds-Fi1 camera, and the panoramic images were performed using the Nis-Elements BR program.

### 2.7. Statistical Analysis

Statistical processing of the obtained data was performed using the SPSS statistics 20.0 application package. The assessment of the statistical significance of differences when comparing groups by quantitative characteristics was carried out using nonparametric methods. The Mann–Whitney test was used to compare the parameters in different groups. The Wilcoxon test was used to assess the statistical significance of differences between values obtained in different timepoints after surgery within the group. Confidence intervals for relative indicators were estimated using the Wilson method. The sample parameters given below have the following designations: Me—median, Q1—upper quartile, Q3— lower quartile, minimum (min) and maximum (max)—minimum and maximum values of the variable, *n*—the size of the analyzed subgroup, *p*-value of the statistical the significance of the differences. The critical significance level was taken equal to 5% (*p* ≤ 0.05).

## 3. Results

### 3.1. Visual and Histological Monitoring of Skin Grafts

**Figure 3:**
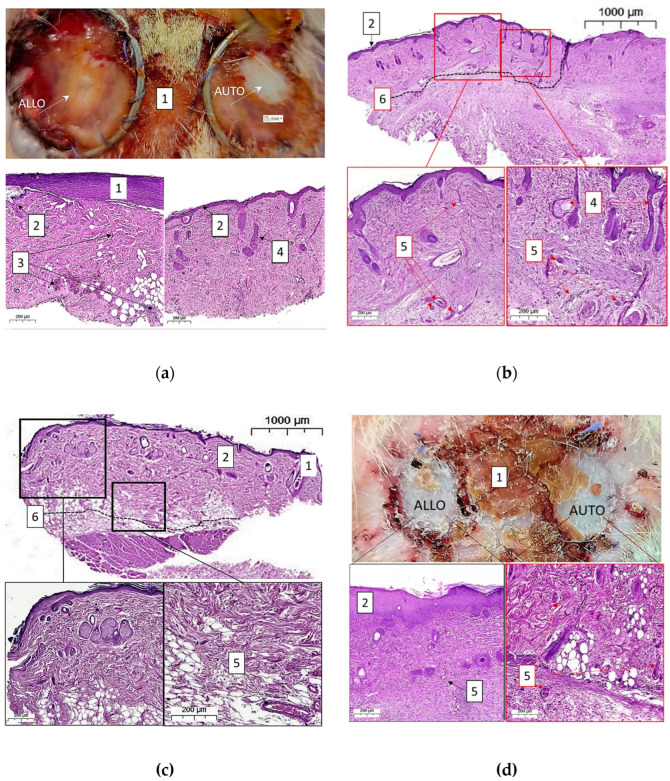
Dynamics of the in vivo macroscopic image and the histological structure of auto- and allografts on the wound from 3 to 13 days after transplantation: 3 days after transplantation (**a**), and 13 days after transplantation (**d**). Histological structure on 6 days after transplantation of an autodermal graft (**b**), and allodermal graft (**c**). 1—epidermis of the near-wound skin; 2—graft epidermis; 3—zones of polymorphic cellular tissue infiltration; 4—skin derivatives; 5—vessels in the graft and wound surrounding tissues; 6—the border between the graft and the recipient wound. Staining of histological specimens with hematoxylin and eosin.

The first group (*n* = 6) of animals had been withdrawn from the experiment 3 days after dermal transplantation. By this time, all allo- and autografts were viable, moderately edematous, and had a pale-cyanotic color. In four animals from the first group, fibrin eschar appeared on the surface of the grafts, and it was more pronounced on allodermal grafts than on autografts. In histological specimens of skin autografts, in contrast to allografts, edema and polymorphic cellular infiltration were weakly expressed, and skin derivatives were preserved (Figure 3a).

In the second, third, and fourth groups of animals, allo- and autodermal grafts retained macroscopic signs of viability in most animals: by the 6th day of observation, one allodermal graft and one autograft were necrotic in the second group of animals. By the 10th day of observation, two allografts and one autograft were necrotic in the third group. In the fourth (final) group, visual signs of the viability of auto- and allodermal grafts were preserved in five animals (Figure 3d); in one case, both grafts remained on the wound but appeared to be necrotic.

On the 6th day after transplantation, moderate neutrophil infiltration and dermal edema were equally expressed in allo- and autografts. However, by this time point, there was a significant difference in the number of microvessels localized in grafts and wound surrounding tissues (Figure 3b,c). By the 10th day of the postoperative period, newly formed vessels appeared in the histological specimens of allodermal and autodermal grafts. By the 13th day of observation, polymorphic cellular infiltration, single newly formed vessels, and skin derivatives were determined. Full-blooded vessels were visualized in the underlying tissues of the recipient wound. In autodermal grafts, the network of newly formed vessels was more pronounced, while the signs of inflammation were pronounced much less. In general, the dynamics of the states of allo- and autografts defined by macroscopic pictures did not differ significantly during 13 days of observation. In viable grafts, visually significant differences were observed only in the early period after transplantation—in the first 3 days after surgery and were expressed in more intense signs of edema and inflammation. At the same time, the histological picture of the microstructure of auto- and allodermal grafts had both similar features and significant differences from 3 to 13 days of the postoperative period: most grafts remained viable, however, inflammatory infiltration and edema of the dermis were more pronounced in allografts, and preserved skin derivatives and newly formed vessels were more common in autodermal grafts.

### 3.2. LDF Monitoring of Skin Grafts

**Figure 4:**
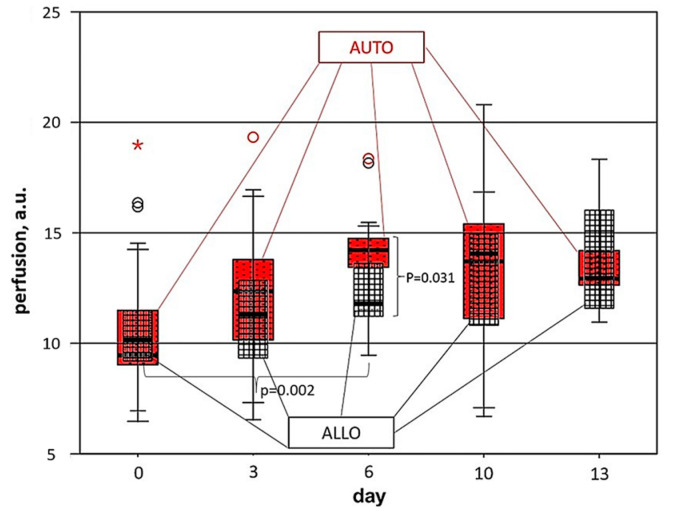
Dynamics of perfusion (a.u.) in allo- and autografts obtained by LDF. A circle is a value out of the Q1–Q3 limits by more than 1.5 interquartile ranges, an asterisk *—more than 3 interquartile ranges.

According to the LDF data (Figure 4), on the first day of transplantation, the perfusion index in the allo- and autografts was similar; however, on the 3rd day after the operation, there was a trend towards an increase in the perfusion index in the autodermal graft. Six days after transplantation, the differences in the intensity of blood circulation in the autodermal and allodermal grafts reached a maximum and became statistically significant: 14.22 [13.43; 14.80] a.u. against 11.81 [11.21; 13.77] a.u. (*p* = 0.031). For autografts, a statically significant difference with initial level (day 0) was revealed on the 6th day after transplantation (*p* = 0.002). This is in accordance with the results of histological analysis showing significant differences in the number of microvessels localized in grafts and wound surrounding tissues (Figure 3b,c) at this timepoint. Starting from the 10th day of postoperative observation, the dynamics of perfusion in grafts became multidirectional: in the allograft, the perfusion index continued to grow, while in the autograft, it began to decrease; by the 13th day, both indicators stabilized at the level of 13 a.u.

### 3.3. DRS Monitoring of Skin Grafts 

**Figure 5:**
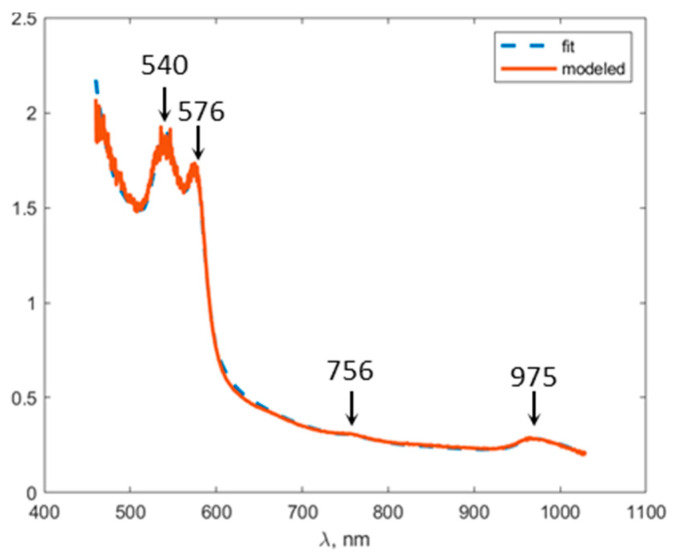
Fitting of a typical extinction spectrum $\mu_{eff}$ (1/mm) obtained experimentally with a model function for skin graft.

An example of fitting an experimentally obtained extinction spectra (2) by a model function (1) composed of absorption (3) and the reduced scattering spectra (4) is presented in Figure 5. The fitting curve reflects the main features of the experimental one: oxyhemoglobin peaks in visible spectral range at 540 and 576 nm, deoxyhemoglobin peak at 756 nm, and water absorption peak at 975 nm.

**Figure 6:**
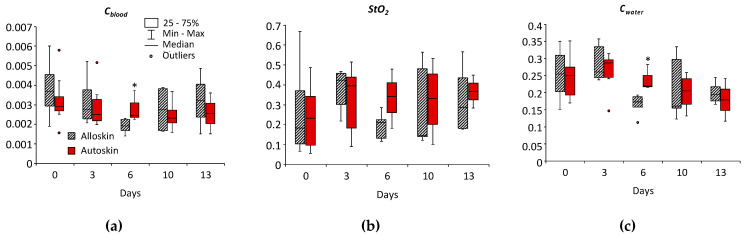
The dynamics of saturation, blood content, $C_{blood}\;\left(a.u.\right)$ (**a**), $StO_{2}$, (**b**) and water content, $C_{water}$, (**c**), reconstructed from the DRS measurements. * statistically significant differences between groups (*p* ≤ 0.05, Mann–Whitney test).

The values of $C_{blood}$, $StO_{2}$, and $C_{water}$ were reconstructed from the measured DRS spectra for allografts and autografts for each rat in each control day according to Table 1. As follows from Figure 6, all reconstructed values in allografts and autografts demonstrate different dynamics in $StO_{2}$, $C_{blood}$, and $C_{water}$. On the 6th day after the transplantation, allografts showed lower values of $C_{blood}$ (*p* = 0.05) and $C_{water}$ (*p* = 0.025) compared to autografts. The differences in blood content revealed by DRS are in agreement with the results of histological analysis showing more blood containing vessels in autodermal grafts as compared to autografts (Figure 3b,c). Blood oxygen saturation in allografts was also less than in autografts, but the differences were not statistically significant. A drop of saturation from 0.38 to 0.19 in allografts on the 6th day (Figure 6b) is presumably connected with the earlier regeneration of vessels that provides blood supply to the autograft. For the same reason, there was a decrease in blood content after allograft transplantation (Figure 6a). The water dynamics in autografts indicate edema as a result of more active inflammation and an increase in the number of vessels. However, no statistically significant changes in the dynamics of investigated parameters were revealed.

### 3.4. OCT Monitoring of Skin Grafts

**Figure 7:**
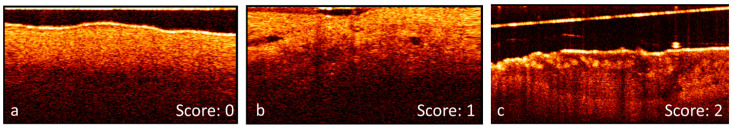
Typical structural OCT-images of grafts corresponding to different edema stages (manifested by low signal areas with pronounced boundaries): (**a**) no edema (score: 0), (**b**) moderate edema (score: 1), and (**c**) strong edema (score: 2). Image size is 3 × 1 mm^2^.

The primary parameters that were monitored using OCT/OCT-A included morphological alterations (primarily presence of edema) and functional response (microcirculatory activity in the superficial graft layers). Typical OCT images of the grafts with different edema stages are shown in Figure 7 together with corresponding scores of edema manifestation for each image in accordance with the developed scoring system. In these images, the edema is manifested by the low signal areas with pronounced boundaries.

**Figure 8:**
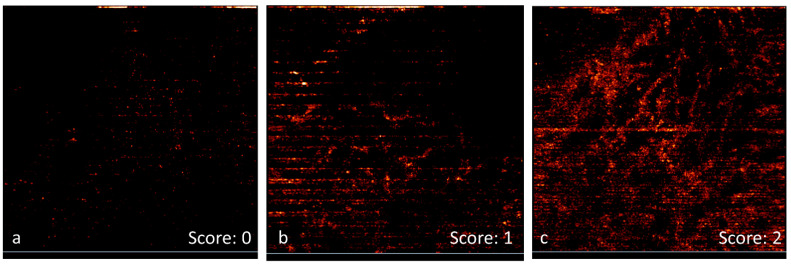
Typical OCT-angiograms of grafts corresponding to different stages of microcirculatory activity: (**a**) no microcirculation (score: 0), (**b**) presence of local microcirculation (score: 1), and (**c**) stable microcirculation with pronounced vessel net (score: 2). Image size is 3 × 3 mm^2^.

**Figure 9:**
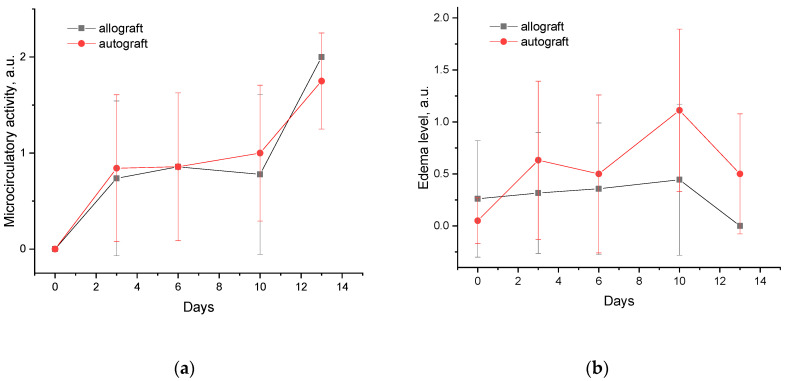
Average dynamics of microcirculatory activity (**a**) and edema (**b**) in allo- and autografts based on OCT diagnostics data.

Figure 8 shows typical OCT angiograms of the grafts with different levels of microcirculatory activity. Figure 8a corresponds to when the microcirculatory activity is absent, which is typical for the first days after transplantation when the blood vessels within the graft are not supplied with blood. Engraftment of the graft leads to the start of the local microcirculatory activity, which corresponds to the image shown in Figure 8b. Further acceptance of the graft leads to an increase in microcirculatory activity accompanied by visualization of the vessel net in OCT-angiogram (Figure 8c). To analyze the observed effect, similar to structural OCT images, we used a semi-quantitative three-grade score to quantify microcirculatory activity based on OCT-A data, and the corresponding scores are shown in Figure 9.

For each animal at each timepoint (0, 3, 6, 10, and 13 days after transplantation), an OCT-study of allo- and autografts was performed with subsequent scoring of the acquired images using the developed scoring system. Despite the semi-quantitative scoring system, for statistical analysis, the scores were treated as quantitative. Figure 9 demonstrates the scores for edema (Figure 9a) and microcirculatory activity (Figure 9b) averaged over all animals available at each timepoint (the number of animals decreased as animals were sacrificed in each timepoint). 

The dynamics of the microcirculation (Figure 9a) demonstrates a monotonous increase in microcirculatory activity for both allografts and autografts, revealing no significant difference between these cases. It is worth noting that no microcirculatory activity is observed on day 0, while on day 13, all the animals left in the experiment demonstrate a high level of microcirculatory activity. This observation is in good agreement with the perfusion dynamics registered by LDF (Figure 4), which also shows the increase in perfusion index during the experiment. The edema dynamics (Figure 9b) also show similar trends for allo- and autografts: an increase in edema level is observed from 0 to 10 days after transplantation followed by a decrease on day 13. Although no statistically significant difference was revealed, it is worth noting that edema manifestations for autografts exceed those for allografts. The general trend in edema level dynamics agrees with the dynamics of water content measured by DRS (Figure 6c), showing an insignificant decrease on the last day of observation.

## 4. Discussion

Skin allograft rejection remains an important cause of postoperative complications and a major limitation to the use of allogeneic skin for wound healing. The terms of allo-dermal graft rejection are difficult to predict; they depend on the immune status of the re-cipient, the ongoing therapy, the immunogenic properties of the graft, the age of the donor and recipient, etc. [26]. In most cases, allogeneic skin grafts are used simultaneously with autologous ones [27]. It should be noted that methods of auto- and allodermoplasty are not competing, since alloskin is intended for temporary closure of a wound defect. For the final closure of the wound after allodermotransplantation (or together with it), tissue engineering technologies or additional methods of processing of donor skin, for example, microtransplantation, are used. In particular, the innovative Rigenera technology makes it possible to obtain a suspension of autologous micrografts with a high regenerative potential [28]. 

At the same time, early (appearing before the development of a complete picture of the immune conflict) objective signs of alteration, specific for allo- and autografts of the skin, have been studied insufficiently. We assumed that the mechanisms of alteration of allo- and autografts have significant differences that can be diagnosed using multimodal optical diagnostics. The results of our work have shown that the simultaneous use of allo- and autoskin micrografts requires non-invasive monitoring of their perfusion, saturation, and edema; critical changes in these parameters can manifest themselves asynchronously.

According to our data, there were no macroscopic signs of necrosis or lysis of auto- and allografts of the skin during the first 3 days after the operation. At the same time, inflammation was more pronounced in allogeneic transplants. At 6-10 days after transplantation, necrosis of a part of the grafts was revealed more in allogeneic than autologous, and on the 10th day, necrosis was more pronounced than on the 6th day. By the 13th day of observation, the proportion of necrotic grafts remained. The absence of obvious signs of allograft rejection within the indicated observation periods is reasonable. The development of a detailed picture of the immune conflict in laboratory rats and mice is variable and occurs from 6 to 30 days after the operation. At the same time, the histological pattern of cellular infiltration of allografts precedes the clinical manifestations of rejection [29].

However, using optical diagnostics tools, we revealed functional and morphological changes in grafts at the early stages of observation. The earliest differences between the allogeneic and autologous transplants were shown for values of blood saturation and blood content obtained by DRS that arise on the 3rd day after the operation and reached a maximum by the 6th day. It should be noted that allografts in these terms were characterized by a lower level of saturation and blood filling, while autografts had more pronounced edema of the dermis. The results of Laser Doppler Flowmetry confirmed the dynamics of DRS data, but with a delay of several days; the maximum differences in tissue perfusion were recorded on the 10th day after transplantation. The earliest signs of changes in the morphological structure of grafts were revealed in vivo using OCT; from the 6th day of observation, a significantly more pronounced edema was detected in the autograft, which confirmed the observations made using DRS. It is possible that the rapidly increasing edema of the autologous skin was the result of intense neovasculogenesis and inflammation in the peri-wound tissues.

The pathogenetic mechanisms of skin allograft necrosis revealed by multimodal optical diagnostics occur in the very early postoperative period and probably include additional mechanisms to that of immune conflict. Primary non-specific inflammatory damage to allografts has been described previously; it is caused by the initiation of polymorphic nuclear cells, macrophages, the release of cytokines, acute phase proteins, and angiogenic factors [30].

In the present study, the animals included in the experiment were carefully selected from different litters, which excludes their closely related status. Nevertheless, the allograft histocompatibility factor was not objectively studied in the frames of this work, and it should be considered when evaluating the results. However, previous studies have shown that the genetic status of the skin allograft has a significant impact on its engraftment/rejection at later stages after transplantation (more than 13 days) [31,32]. So, we believe that the abovementioned analysis was not essential in the present study; however, it would be beneficial in more prolonged monitoring.

## 5. Conclusions

Skin allografts undergo immune rejection, and the terms of rejection are very variable. Non-invasive optical diagnostic tools revealed changes in the morphological microstructure, the rate of restoration of blood circulation, and oxygen exchange in the early stages after transplantation into the recipient wound, specific for the allo- and autograft. The earliest differences between the allogeneic and autologous transplants were revealed using DRS; the differences in blood content reached 14% (*p* = 0.05) by day 6. The results of LDF corresponded to the DRS data with a delay of several days; on the 10th day after transplantation, perfusion in allografts was lower by 17% than in autografts (*p* = 0.03). The earliest signs of changes in the morphological structure of the grafts were revealed in vivo using OCT; from the 6th day of observation, a significantly pronounced edema was detected in the autograft, which correlates with the DRS and histological data.

## Figures and Tables

**Table 1:** Skin engraftment experiment design.

Day after Thermal Burn	−3	0	3	6	10	13
Surgical procedure	Burn 20 %	partial fascial necrectomy; allo- and autografting	Dressing; breeding of 6 animals (1 group)	Dressing; breeding of 6 animals (2 group)	Dressing; breeding of 6 animals (3 group)	Dressing; breeding of 6 animals (4 group)
Examination procedure		LDF, OCT, DRS	LDF, OCT, DRS; Biopsy in group 1	LDF, OCT, DRS; Biopsy in group 2	LDF, OCT, DRS; Biopsy in group 3	LDF, OCT, DRS; Biopsy in group 4

**Table 2:** Semi-quantitative scoring system for the classification of OCT-images.

Edema Score	Edema Manifestation	Features of Structural OCT Image
0	No edema	No low signal level areas
1	Weak edema	Single small-scale low signal level inhomogeneities within a single OCT cross-section
2	Pronounced edema	Large scale low signal level inhomogeneities and/or numerous low signal level inhomogeneities within a single OCT cross-section
Microcirculatory activity score	Microcirculatory system state	Features of OCT-angiography image
0	No microcirculation	No signal
1	Local microcirculation	Visualization of single vessels in the field of view
2	Normal microcirculation	Visualization of a developed vessel net

## Data Availability

The data used in this research is available from the corresponding author upon reasonable request.
